# Residual tumor after the salvage surgery is the major risk factors for primary treatment failure in malignant ovarian germ cell tumors: A retrospective study of single institution

**DOI:** 10.1186/1477-7819-9-123

**Published:** 2011-10-11

**Authors:** Chung Won Lee, Min Jong Song, Sung Taek Park, Eun Young Ki, Sung Jong Lee, Keun Ho Lee, Ki Sung Ryu, Jong Sup Park, Soo Young Hur

**Affiliations:** 1 Department of Obstetrics and Gynecology, Yeouido St. Mary's Hospital, School of Medicine, the Catholic University, Yeouido-dong, Yeodeungpo-gu, Seoul, 150-713, Korea; 2 Department of Obstetrics and Gynecology, Daejeon St. Mary's Hospital, School of Medicine, the Catholic University, 64, Daeheung-dong, Jung-gu, Daejeon, 301-723, Korea; 3 Department of Obstetrics and Gynecology, Seoul St. Mary's Hospital, School of Medicine, the Catholic University, 505, Banpo-dong, Seocho-gu, Seoul, 137-040, Korea; 4 Department of Obstetrics and Gynecology, St. Vincent Hospital, School of Medicine, the Catholic University, 93-6, Ji-dong, Paldal-gu. Geonggy-do, 442-723, Korea

**Keywords:** Malignant ovarian germ cell tumor, Primary treatment failure, Prognostic factors

## Abstract

**Background:**

Malignant ovarian germ cell tumors are rare, and knowledge of their prognostic factors is limited, with little available randomized data. This study was conducted to evaluate the clinicopathologic characteristics of malignant ovarian germ cell tumors and to determine the association of their prognostic factors to primary treatment failure.

**Methods:**

The medical records of 57 patients with stages I to IV malignant ovarian germ cell tumor were retrospectively reviewed, and their clinicopathologic and treatment-related data were collected and analyzed.

**Results:**

The median age at the diagnosis was 23.3 years (range: 8-65 years), and the median follow-up period was 108 months (range: 48-205 months). The histological types of the tumors were immature teratoma (n = 24), dysgerminoma (n = 20), endodermal sinus tumor (n = 8), mixed germ cell tumor (n = 4), and choriocarcinoma (n = 1). 66.7% of the patients had stage I disease; 5.2%, stage II; 26.3%, stage III; and 1.8%, stage IV. After the initial surgery, 49 patients (86%) received cisplatin-based chemotherapy. The five-year survival rate was 96.5%. There were six primary treatment failures, with two of the patients dying of the disease, and the median time to the recurrence was 8 months. The histological diagnosis (*P *< 0.0001), tumor stage (*P *= 0.0052), elevation of beta-hCG (*P = *0.0134), operation methods (*P = *0.0006), and residual tumor after the salvage surgery (*P *< 0.0001) were significantly associated with the risk of primary treatment failure in the univariate analysis. In the multivariate analysis, the residual tumor after the salvage surgery was the only significant variable associated with primary treatment failure (*P *= 0.0011, Hazard ratio = 29.046, 95% Confidence interval 3.832-220.181).

**Conclusion:**

Most malignant ovarian germ cell tumors have excellent prognoses with primary treatment, and good reproductive outcomes can be expected. Because primary treatment failure is associated with the residual disease after the salvage surgery, knowledge of the presence or absence of this risk factor may be helpful in risk stratification and individualization of adjuvant therapy in malignant ovarian germ cell tumors. Further large-scale prospective studies to confirm these results should be performed.

## Background

Malignant ovarian germ cell tumors (MOGCTs) are a heterogeneous group of tumors that have several histologically different types derived from primordial germ cells of the embryonic gonad [1]. In contrast to epithelial ovarian cancer (EOC), these tumors are rare cancers, accounting for less than 5% of all ovarian malignancies, and typically occur primarily in girls and young women [2]. In addition, 50-75% of MOGCTs are stage I at their diagnosis; and even in the advanced stage of the disease, the survival rates are 60-80% [2-4]. In the past, radical surgery was performed due to the presumed risk of microscopic involvement of the grossly normal contralateral ovary and uterus; but recent multimodality therapy with initial fertility-sparing surgery and platinum-based chemotherapy has improved the prognosis significantly, even in patients with advanced diseases.

Because of the rare incidence of these tumors, however, experience with MOGCTs is limited, with little available randomized data and information on their management in a sufficient number of patients [1, 4-5]. There is no doubt that early-stage MOGCT patients have excellent prognoses with standard treatment, whereas advanced and recurrent tumors remain a challenge. In addition, the fact that most MOGCTs occur in reproductive-age women is of great importance to the treatment of the disease. Prognostic factors are very important because they may influence the decision to perform less radical surgery in the early stages of the disease without compromising fertility and may be helpful in creating risk stratification models for individualization of adjuvant therapies [4,6]. This study reports the experience of an institution in the management of MOGCTs over a period of about 20 years. The aim of this study was to evaluate the clinicopathologic characteristics of MOGCTs and to determine the association of their prognostic factors to their primary treatment failure.

## Methods

After obtaining approval from the Institutional Review Board (IRB), all women diagnosed with histologically proven MOGCTs were identified using our institutional medical database. Patients who were primarily treated elsewhere and were referred to the authors' institution for adjuvant chemotherapy or salvage therapy after the recurrence of their disease were excluded. The medical records of the patients were retrospectively reviewed, and data were collected regarding their demographics, disease characteristics, management patterns, disease recurrence, and survival.

The histological types of the disease were classified according to the World Health Organization (WHO) System [7], and the stages were assigned as described by the International Federation of Gynecology and Obstetrics (FIGO). All the patients underwent maximal cytoreductive surgery as their primary treatment, and depending on their pathologic results and operative findings, some received adjuvant chemotherapy according to institutional guidelines. The initial surgery was considered a staging operation when peritoneal cytology, multiple biopsies, infracolic omentectomy, and lymphadenectomy were also performed. A fertility-sparing surgery was defined as preservation of the uterus and at least part of one ovary to preserve fertility. The duration of the follow-up was calculated from the pathologic diagnosis to the date of the last visit. The time to recurrence was defined as the period from the last date of primary treatment (the date of surgery or the date of adjuvant chemotherapy) to the first observation of disease progression. The recurrence/progression free survival and the time to recurrence were estimated using the method of Kaplan and Meier, and the log rank test with SPSS (Version 12.0; SPSS, Inc., Chicago, Ill) was used to assess the statistical significance of the prognostic factors. Multivariate analysis was performed using Cox's regression analysis. P values that were less than 0.05 were considered statistically significant. All the P values were two-sided.

## Results

### Clinicopathologic characteristics and treatment methods

Seventy-two women who were treated in the Division of Gynecologic Oncology at Seoul St. Mary's Hospital between January 1990 and July 2009 were identified as having had MOGCT. Among them, 57 patients received primary treatment in the authors' institution, and the remaining 15 patients were primarily treated elsewhere and were referred to the authors' institution for adjuvant chemotherapy (n = 8) or salvage therapy after the recurrence of their disease (n = 7). The clinicopathologic characteristics and treatment methods of 57 patients are summarized in Tables 1 and 2. The median age at the diagnosis was 23.3 years (range: 8-65 years), and the median follow-up period of the survivors was 108 months (range: 48-205 months). Most of the patients were nulliparous (n = 45, 78.9%). The tumor size ranged from 4 to 25 cm in the maximal diameter, and the mean tumor diameter was 15.2 cm. The histological types of the tumors were immature teratoma (n = 24), dysgerminoma (n = 20), endodermal sinus tumor (EST) (n = 8), mixed germ cell tumor (n = 4), and choriocarcinoma (n = 1). Most of the tumors were unilateral (n = 51, 89.4%), but the histological types of bilateral tumors were dysgerminoma (3/6, 50%), EST (2/6, 33.3%), and mixed germ cell tumor (1/6, 16.7%). 66.7% of the patients had stage I disease; 5.2%, stage II; 26.3%, stage III; and 1.8%, stage IV. The alpha-fetoprotein (AFP) levels were known for 46 patients, and two patients with immature teratoma and five with EST had elevated AFP levels that were higher than 100 ng/ml. Three patients with mixed germ cell tumor who had elements of EST also had elevated AFP levels. The beta-human gonadotrophic hormone (hCG) was checked in 53 patients, and was elevated in four patients with dysgerminoma, in three with mixed germ cell tumor, and in one with choriocarcinoma.

**Table 1:** The clinicopathologic factors associated with recurrence/progression in MOGCTs

Variables	No. Patients (%)	Recurrence No (Rate, %)	P value
Age, years			
< 20	26(45.6)	2(3.5)	0.1177
20-40	28(49.1)	3(5.3)	
> 40	3(5.3)	1(1.8)	
Histological diagnosis			
Dysgerminoma	20(35.1)	0(0.0)	< 0.0001
Immature teratoma	24(42.1)	1(1.8)	
Endodermal sinus tumor	8(14.0)	2(3.5)	
Choriocarcinoma	4(7.0)	2(3.5)	
Mixed germ cell tumor	1(1.8)	1(1.8)	
Histological diagnosis^a^			
Dysgerminoma	20(35.1)	0(0.00)	0.0716
Non-dysgerminoma	37(64.9)	6(10.5)	
Stage			
I	38(66.7)	1(1.8)	0.0052^b^
II	3(5.2)	0(0.0)	
III	15(26.3)	4(7.0)	
IV	1(1.8)	1(1.8)	
Volume of ascites, cc			
< 100	33(57.9)	2(3.5)	0.1277
≥ 100	24(42.1)	4(7.0)	
Elevated AFP, ng/ml (n = 46)			
< 100	36(78.3)	4(8.7)	0.4089
≥ 100	10(21.7)	2(4.4)	
Beta-hCG (n = 53)			
Normal	45(84.9)	3(5.7)	0.0134
Elevated	8(15.1)	3(5.7)	

AFP, alpha-fetoprotein; hCG, human gonadotrophic hormone.

^a ^Comparison between two groups.

^b ^Comparison between stage I-II and stage III-IV.

**Table 2:** Analyses of treatment-related variables associated with recurrence/progression in MOGCTs

Variables	No. Patients (%)	Recurrence No (Rate, %)	P value
Treatment modalities			
Surgery only	8(14.0)	1(1.8)	0.4342
Surgery + chemotherapy	49(86.0)	5(8.8)	
Operation methods			
Fertility sparing	42(73.7)	1(1.8)	0.0006
Cystectomy without staging op.	1(1.8)	0(0.0)	
USO without staging op.	20(35.1)	0(0.0)	
USO with staging op.	21(36.8)	1(1.8)	
Non-fertility sparing	15(26.3)	5(8.8)	
Hysterectomy + BSO without staging op.	5(8.8)	0(0.0)	
Hysterectomy + BSO with staging op.	10(17.5)	5(8.8)	
Chemotherapy regimens (n = 49)			
BEP	9(18.4)	1(2.0)	0.9008
EP	28(57.1)	3(6.1)	
VBP	2(4.1)	0(0.0)	
Others	10(20.4)	10(20.4)	
Residual lesions			
None	47(82.5)	1(1.8)	< 0.0001
≤ 1 cm	8(14.0)	3(5.3)	
> 1 cm	2(3.5)	2(3.5)	

USO, unilateral salpingo-oophorectomy; BSO, bilateral salpingo-oophorectomy; BEP, bleomycin, etoposide, and cisplatin; EP, etoposide and cisplatin; VBP, vinblastine, bleomycin, and cisplatin.

Regarding the surgery for the primary tumor, a fertility-sparing surgery was performed in 42 patients (73.7%), a staging operation in 31 patients (54.4%). Lymph node involvement was reported in 10 of the 31 patients who underwent the staging operation. Most of the patients who underwent surgery had no residual disease (n = 47, 82.5%); but based on the pathologic results and operative findings, stage I dysgerminoma or stage Ia, grade 1 immature teratoma was observed without postoperative adjuvant chemotherapy, and the patients who had the other pathologic results and operative findings were allowed to postoperative adjuvant chemotherapy. Forty nine patients underwent postoperative cisplatin-based chemotherapy, and the most common regimen was etoposide and cisplatin (EP), followed by bleomycin, etoposide, and cisplatin (BEP) (Table 2). Eight of the patients had pelvic or abdominal residual disease with a tumor diameter of less than 1 cm; one patient had a 2 cm metastasis on his liver surface; and the patient who was diagnosed with choriocarcinoma had unresectable lung metastasis.

### Prognostic factors associated with primary treatment failure

After a median follow-up of 108 months (range: 48-205 months), there were six primary treatment failures, with two patients dying of the disease, and the five-year survival rate was 96.5%. Recurrence was observed in five patients (8.8%), and disease progression was observed in one patient (1.8%). The time to recurrence ranged from 4 to 12 months, and the median time to recurrence was 8 months. The detailed clinical features and outcomes of the patients who experienced primary treatment failure are summarized in Table 3.

**Table 3:** Summary of clinicopathologic features, treatment, and outcome of those who failed primary treatment

Pt's ID	Age	Histology/Stage	Primary treatment	Outcome of primary treatment	Time to relapse (months)	Site of Relapse/ progression	Salvage treatment	Outcome
1	18	Choriocarcinoma/ Stage IV	TAH + BSO + staging op + BEP chemo.	CR	4	Brain	EMA-CO chemo.	DOD
2	25	Immature teratoma/ Stage Ic	USO + staging op	CR	11	PAN	BEP chemo	NED at age 33
3	22	Mixed germ cell tumor/Stage IIIa	TAH + BSO + staging op + EP chemo	CR	12	Mesentery	EP chemo	NED at age 33
4	19	EST/Stage IIIa	TAH + BSO + staging op + CP chemo.	CR	7	Mesentery	EP chemo.	NED at age 29
5	29	EST/Stage IIIc	TAH + BSO + staging op + EP chemo.	CR	6	Pelvic lymph node	VBP chemo.	NED at age 36
6	65	Mixed germ cell tumor/Stage IIIc	TAH + BSO + staging op + EP chemo	Progressi-on		Liver	Cisplatin + Paclitaxel chemo.	DOD

TAH, total abdominal hysterectomy; BSO, bilateral salpingo-oophorectomy; USO, unilateral salpingo-oophorectomy; EMA-CO, etoposide, methotraxate, actinomycin-D, vincristine, and cyclophosphamide; BEP, bleomycin, etoposide, and cisplatin; EP, etoposide and cisplatin; CP, cisplatin and cyclophophamide; VBP, vinblastine, bleomycin, and cisplatin; CR, complete remission; PAN, paraaortic lymph node; DOD, died of disease; NED, no evidence of disease.

**Figure 1:**
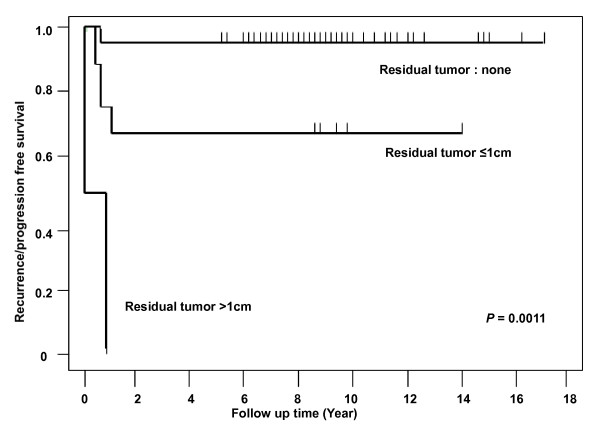
**The recurrence/progression free survival based on the residual tumor after the salvage surgery**. Figure 1 presents the Kaplan-Meier estimate of the recurrence/progression free survival based on the residual tumor after the salvage surgery (no residual mass *vs*. ≤ 1 cm of residual mass *vs*. > 1 cm of residual mass). The one-year recurrence/progression-free survival rate of the patients with no residual tumor after their salvage surgery was 97.8%, and only 62.5% for the patients with ≤ 1 cm of residual mass, and 0% for the patients with > 1 cm of residual mass.

The histological diagnosis (*P *< 0.0001), tumor stage (*P *= 0.0052), elevation of beta-hCG (*P = *0.0134), operation methods (*P = *0.0006), and residual tumor after the salvage surgery (*P *< 0.0001) were significantly associated with the risk of primary treatment failure in the univariate analysis **(**Tables 1 and 2). The recurrence/progression-free survival rate was 100% for dysgerminoma (20/20), 95.8% for immature teratoma (23/24), 75.0% for EST (2/8), 50.0% for mixed germ cell tumor (2/4), and 0% for choriocarcinoma (*P *< 0.0001). The rate of primary treatment failure was 1.8% for women in stages I and II, and 8.8% for women in stages III and IV (*P *= 0.0052). Complete tumor resection was performed on 47 patients and showed a lower recurrence rate than incomplete resection (*P *< 0.0001). The age, volume of ascites, elevation of AFP, treatment modalities, and chemotherapy regimen did not affect the primary treatment failure. In the multivariate analysis, the residual tumor after the salvage surgery was the only significant variable associated with the primary treatment failure with respect to the MOGCTs (*P *= 0.0011, Hazard ratio [HR] = 29.046, 95% Confidence interval [CI] 3.832-220.181). The Kaplan-Meier estimate of the recurrence/progression free survival based on the residual tumor after the salvage surgery (no residual mass *vs*. ≤ 1 cm of residual mass *vs*. > 1 cm of residual mass) is shown in Figure 1. The one-year recurrence/progression-free survival rate of the patients with no residual tumor after the salvage surgery was 97.8%, and only 62.5% for the patients with ≤ 1 cm of residual mass, and 0% for the patients with > 1 cm of residual mass.

During the follow-up period, 14 patients reported attempting to conceive post-treatment, and seven patients had 10 successful pregnancies with 10 full-term live births. All these seven women had received adjuvant EP or BEP chemotherapy. There is no evidence of birth defects or other disabilities in any of the offspring. One patient diagnosed to stage IIIc EST and received 4 cycles of EP chemotherapy had acute myeloid leukemia (AML) 19 months after starting EP chemotherapy with 5-day etoposide (100 mg/m^2 ^on days 1-5), cisplatin (100 mg/m^2 ^on days 1), and a cumulative dose of etoposide was 3200 mg/m^2^, but the patient received allogenic bone marrow transplantation, and has no evidence of disease at present.

## Discussion

The introduction of adjuvant platinum-based chemotherapy in cytoreductive surgery has improved the prognosis of MOGCTs, and the overall disease-free survival rate is more than 95% [8]. The occurrence of most MOGCTs in reproductive-age women requires special consideration, however, as it means most MOGCT patients must be treated with a fertility-sparing modality that does not compromise survival.

To accomplish this therapeutic intent, the most important thing to do is risk stratification of the diseases according to their prognosis. With their limited number of cases, however, virtually no randomized controlled trials have been conducted on MOGCTs, and several different, inconsistent prognostic factors have been reported in retrospective studies. A study by Lai et al. analyzed the prognostic factors of 93 MOGCTs, and the authors suggested that the histology (dysgerminoma *vs*. non-dysgerminoma, *P *< 0.0001) and the FIGO stage (I-II *vs*. III-IV, *P *= 0.001) were significantly associated with treatment failure, whereas the histology (dysgerminoma *vs*. non-dysgerminoma, *P *< 0.0004), the residual tumor after the salvage surgery (≥ 1 cm *vs*. < 1 cm, *P *= 0.0014), and the salvage high-dose chemotherapy failure (*P *= 0.0405) significantly influenced the overall survival [9]. In a more recent study, Lee et al. reported that the age (≤ 40 years *vs*. > 40 years, *P *= 0.031) and the FIGO stage (I-II *vs*. III-IV, *P = *0.032) significantly predicted recurrence in 196 Korean women with MOGCTs [4]. In the study of Murugaesu et al., univariate and multivariate analyses of 113 MOGCTs demonstrated the importance of tumor markers (AFP and beta-hCG) (relative risk [RR], 3.90; *P *= 0.009) and the initial stage of the disease (RR, 5.96; *P *= 0.03) as predictors of overall survival, whereas the age at the diagnosis had no prognostic value [10]. In still another study, Kumar et al. suggested lymph node involvement as an independent predictor of poor survival, with a hazards ratio of 2.87 (95% CI 1.439-5.725; *P *< 0.05) [6]. Several studies on non-dysgerminomatous ovarian germ cell tumors (NDOGCTs) have also been reported [11-14]. In these studies, an early stage [11-13], minimal residual tumor after the initial surgery [11-13], less than 100 cc of ascites [11,12], platinum-based chemotherapy [11,12], an AFP level less than 1,000 kU/L, and a non-EST histology were found to be significantly related to a more favorable prognosis [14]. In conclusion, taking into account the results from largest series reported on MOGCTs, stage, residual disease, histological type, elevation of tumor markers, and chemotherapeutic regimen seem to be recognized as prognostic factors for patients with MOGCT. In this study, the histological diagnosis (*P *< 0.0001), tumor stage (*P *< 0.0001), elevation of beta-hCG (*P = *0.0134), operation methods (*P = *0.0006), and residual tumor after the salvage surgery (*P *< 0.0001) were found to be significantly associated with the risk of primary treatment failure in the univariate analysis. In the multivariate analysis, however, the residual tumor after the salvage surgery was the only significant variable associated with primary treatment failure for MOGCTs (*P *= 0.0011, HR = 29.046, 95% CI 3.832-220.181), whereas the correlation of the other factors could not be proven (data not shown). Significant P-value with a very wide confidence interval of this factor may have been caused by the fact that the majority of MOGCTs are so early staged that maximal cytoreductive surgery can be performed in most cases. In other words, it may have been caused by relatively small size of the group with residual lesions due to the excellent surgical outcome of the MOGCTs. Type of operation (fertility sparing *vs*. non-fertility sparing operation) was found to be significantly associated with the risk of primary treatment failure in the univariate analysis (*P = *0.0006), but not in the multivariate analysis (*P *= 0.1493). The major reasons to perform a non-fertility sparing operation instead of a fertility sparing operation in this study are the willingness of patients to preserve fertility and the extent of disease. Because the authors have the conviction that stage III-IV or unresectable MOGCTs are expected to have poor prognostic outcome according to the authors' clinical experiences, more radical operations may be performed in this group. Therefore, patients who had non-fertility sparing operations seemed to have poor prognosis in the univariate analysis, but type of operation was not actually significant in the statistics.

The principles of cytoreductive surgery in epithelial ovarian cancer, i.e., resection of as much gross tumor as possible, have been applied to the management of MOGCTs. Two Gynecologic Oncology Group (GOG) studies indicated that patients with unresectable or incompletely resected MOGCTs had fewer chances of full remission after chemotherapy than patients with minimal residual disease [15,16]. Both studies had limited sample sizes, however, and the chemotherapy regimens that they employed (VAC, vincristine, actinomycin D, and cycolphosphamide; and PVB, cisplatin, vinblastine, and bleomycin, respectively) are not the most active regimens at present [8]. The role and extent of cytoreductive surgery remain controversial despite its routine use, unless the randomized data can prove the therapeutic advantages. Nevertheless, taking into account the results of many series [9,11-13,15,16] and the chemotherapy-sensitive nature of the disease, an adequate attempt at maximal cytoreduction without compromising future fertility seems a reasonable surgical approach at present. Many studies have shown that fertility-sparing surgery is a safe, reasonable treatment option that does not compromise survival, but support for such an approach shall arise not from prospective randomized trials but from experiential evidence. Gershenson et al., and Kurman et al. did not observe worsening of the prognosis associated with unilateral salpingo-oophorectomy (USO) in MOGCT patients, compared to BSO [2,17]. Tewari et al. reported that the prognoses of 46 patients with MOGCTs who underwent USO and postoperative systemic chemotherapy were excellent (five-year survival rate, 93%) [18]. In this study, of the 42 patients with MOGCTs who underwent fertility-sparing surgery, only one grade I immature teratoma patient who did not undergo postoperative adjuvant chemotherapy had a recurrence in her para-aortic lymph node, and was successfully salvaged with BEP chemotherapy.

The introduction of multimodality therapy with initial surgery and subsequent chemotherapy has dramatically improved the prognosis of patients with MOGCTs. The evolution of systemic therapy of MOGCTs has paralleled the advances made in the treatment of testicular germ cell tumors [1]. Lessons learned from randomized clinical trials in testicular cancer cases have been applied to the much fewer ovary tumors. VAC was the first efficient chemotherapy regimen to achieve about 50% remission in advanced stages, and up to 85% in early stages in the treatment of NDOGCTs [15,19]. The inclusion of cisplatin in the systemic therapy of MOGCTs (PVB and BEP) has proven to yield a better chance of cure of up to 60-80%, even with incompletely resected advanced-stage tumors [8,16,20-23]. The largest prospective study of BEP as adjuvant therapy was performed by GOG [23]. In the study, of 93 patients who had MOGCT and underwent three courses of BEP post-operatively, 89 patients have remained free of the disease (89/93, 96%). A multi-institutional trial reported that the BEP regimen showed equal efficacy and less toxicity than the PVB regimen in patients with testicular cancer [24], and therefore, the BEP regimen is regarded as the standard regimen for first-line chemotherapy of MOGCTs. In this study, the most common chemotherapeutic regimen selected was EP (n = 28) rather than BEP (n = 8). While a randomized study has found that three cycles of the BEP regimen are superior to the EP regimen in the case of testicular cancer (relapse-free survival rate, 84% *vs*. 69%, *P *= 0.03) [25], because of the authors' terrible experiences in fatal pneumonitis associated with bleomycin, we selected the EP regimen for most of their patients with MOGCTs. As mentioned, the chemotherapy regimen was not influenced by the prognosis of MOGCTs, and the EP regimen was as highly effective as the BEP regimen in this study.

Given the prognosis and age of most of the patients, the important things to consider are the long-term side-effects of chemotherapy. The most serious potential long-term forms of toxicity of chemotherapy are secondary malignancies, foremost of which is acute non-lymphoblastic leukemia. This is a well-known complication of relatively long-term alkylating agent therapy. In the study of Pedersen-Bjergaard et al., they described the mean cumulative risk of leukemic complications in 212 patients with germ cell tumors treated with BEP increased steadily from 15 months after start of etoposide and reached a mean of 4.7% at 5.7 years, and compared with the risk in the general population, the relative risk of overt leukemia was 336 [26]. The authors encountered a case of AML development in one patient 19 months after she started the etoposide-containing regimen (1/36, 2.8%), but the patient underwent allogenic bone marrow transplantation, and has no evidence of disease at present. Another important issue is the impact of chemotherapy on the reproductive function. According to a review of Gershenson, 27 of 40 patients who underwent fertility-sparing surgery maintained regular menses after their completion of chemotherapy, 33 had regular menstruation at the time of their follow-up, and of the 16 patients who had attempted to become pregnant since completing the chemotherapy, 11 delivered 22 healthy infants without major birth defects [27]. In a questionnaire-based study by Tangir et al., they reported that of 64 patients who underwent fertility-sparing surgery, 38 attempted conception and 29 became pregnant (76%) with no congenital anomalies [28]. In this study, of the 14 patients who attempted to conceive after their treatment, 50% had successful pregnancies with no evidence of birth defects or other disabilities in any of their offspring.

This report was limited by its small number of cases due to the rarity of the disease, and the retrospective study design. This report has highlighted, however, the current state of clinicopathology, management, and prognosis of MOGCTs. In summary, this report showed that most MOGCTs have excellent prognosis with primary treatment, and good reproductive outcomes can be expected for them. Because the primary treatment failure is associated with the residual disease after the salvage surgery, knowledge of the presence or absence of this risk factor may be helpful in risk stratification and individualization of adjuvant therapy in MOGCTs. Further large-scale prospective studies to confirm these results are needed.

## Conclusions

This study was undertaken to evaluate the clinicopathologic characteristics of MOGCTs and to determine the association of their prognostic factors to primary treatment failure in female patients. Residual tumor after the salvage surgery was found to be the only risk factor for primary treatment failure of MOGCTs, but most MOGCTs have excellent prognosis with primary treatment, and good reproductive outcomes can be expected for them. Further studies are required to determine the most effective management strategies in patients with these risk factors for primary treatment failure.

## Competing interests

The authors declare that they have no competing interests.

## Authors' contributions

SYH, the corresponding author of this study, provided the major idea, planed and approved the written work. CWL contributed mainly in designing, literature review and writing the manuscript. Both STP and MJS collected the clinicopathologic data, and described all findings. EYK and SJL gave an important help for statistical analyses. KHL, KSR, and JSP gave advices about clinical variables to analyze and edited the discussion. All authors read and approved the manuscript.
